# Predictive Value of the Lowest Serum Albumin Level During Hospitalization in Patients With Intracerebral Hemorrhage

**DOI:** 10.1155/nri/7248981

**Published:** 2026-06-26

**Authors:** Biao Zhao, Hua-Zhen Zhang, Tao Liu, Dan Liu, Da-Wei Wang

**Affiliations:** ^1^ Department of Neurosurgery, The Second Affiliated Hospital of Bengbu Medical University, Bengbu, 233002, China; ^2^ Department of Neurosurgery, Xuanwu Hospital, Capital Medical University, Beijing, 100053, China, ccmu.edu.cn; ^3^ Department of Endocrinology, The Second Affiliated Hospital of Bengbu Medical University, Bengbu, 233002, China

**Keywords:** albumin, Glasgow Coma Scale, hematoma volume, intracerebral hemorrhage, predictor, the lowest serum albumin

## Abstract

**Background and Purpose:**

This study aimed to explore the potential association between the lowest serum albumin concentration during hospitalization and the prognosis of patients with intracerebral hemorrhage (ICH) in order to provide a scientific basis for selecting and optimizing clinical treatment options.

**Methods:**

A total of 268 patients with ICH were retrospectively analyzed. The study outcome was 90‐day mRS (mRS > 2). Univariate and multivariate analyses and ROC curve analyses were used to identify the correlation between the lowest serum albumin concentration and the prognosis of patients with ICH.

**Results:**

(1) The lowest serum albumin concentration, Glasgow Coma Score (GCS), and hematoma volume were independent predictors of outcome in patients with ICH (*p* < 0.05). (2) A good outcome in patients with ICH was predicted when the lowest serum albumin levels were > 31.8 g/L (AUC = 0.849, 95% CI: 0.801–0.890, *p* < 0.001), the GCS was > 11 (AUC = 0.878, 95% CI: 0.833–0.915, *p* < 0.001), and the hematoma volume was ≤ 23.6 mL (AUC = 0.863, 95% CI: 0.815 to 0.902, *p* < 0.001). (3) The lowest serum albumin concentration was statistically different between GCS (3–8, 9–12, 13–15: 29.30 [3.90], 33.80 [6.85], 36.70 [5.55]) (*χ* ^2^ = 69.696, *p* < 0.001) and had a positive correlation with GCS (*r*_*s*_ = 0.569).

**Conclusions:**

The lowest serum albumin concentration during hospitalization was an independent predictor of poor outcome in ICH, and when serum albumin was greater than 31.8 g/L, it predicted a good outcome. In addition, the degree of consciousness disturbance may have some influence on the serum albumin level, and the specific mechanism needs further study.

## 1. Introduction

Cerebrovascular diseases seriously endanger human health globally. The rising incidence in China is showing a worrying trend. Among the cerebrovascular diseases, intracerebral hemorrhage (ICH) is a neurological disease that requires urgent attention due to its high incidence and disability rate [1]. ICH, as a type of stroke classification, accounts for about 10%–20% of all stroke cases, but its mortality is as high as 40%–50%, a figure that highlights the serious threat that ICH poses to the life and health of patients [2]. In addition, ICH causes great pain to the patients and also imposes a heavy economic burden on families and society [3].

Therefore, exploring the prognostic factors of ICH is crucial for improving patients’ quality of life and reducing the burden of disease. According to several studies, low serum albumin levels have been shown to be an independent predictor of increased morbidity and mortality in ICH and subarachnoid hemorrhage [4–7]. However, the albumin measurements used in these studies were taken at admission only, which limits their value for guiding treatment decisions. Albumin levels are dynamic indicators influenced by multiple factors such as inflammatory responses, nutritional status, and disease severity. The lowest value during hospitalization may better reflect the patient’s critical stress state and overall physiological reserve compared to a single admission measurement. But existing studies have primarily focused on albumin levels at admission. Given that albumin levels are dynamic during the acute phase, current research lacks evidence regarding whether their lowest values during hospitalization provide prognostic value beyond baseline measurements or serve as novel therapeutic targets for dynamic management. Therefore, this study aimed to analyze the factors influencing the prognosis of patients with cerebral hemorrhage, especially the role of the lowest serum albumin level, and explore their potential clinical application value.

## 2. Methods

### 2.1. Patient Selection

**FIGURE 1:**
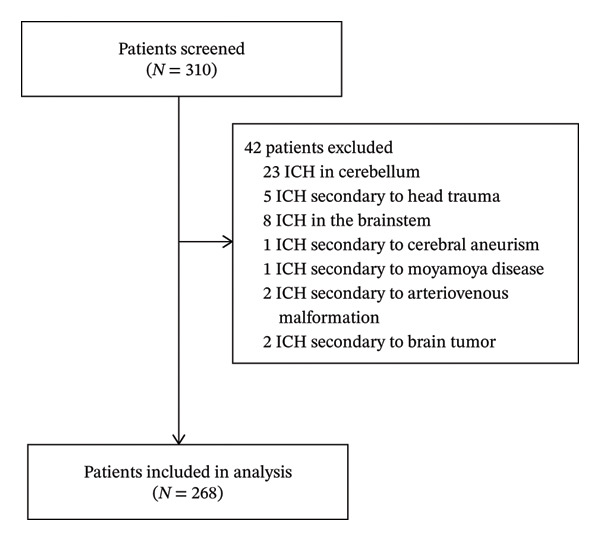
Flowchart showing the selection process for the study cohort.

We included patients who were admitted to our hospital between February 2020 and December 2023 with ICH, as confirmed by brain CT. Patients were eligible for inclusion if they had been diagnosed with ICH by CT, their clinical records were complete, the preonset Modified Rankin Scale (mRS) score was less than or equal to the score of 2 points, and patients were over 18 years of age. Patients were not eligible if the hemorrhage was secondary to a brain tumor, cerebral aneurism, arteriovenous malformation, moyamoya disease, or head trauma, or the hematoma was located in the brainstem and cerebellum, or the brain CT showed artifacts that could influence the calculation of the hematoma volume, or patients with incomplete data. Finally, the analysis was performed on 268 patients (Figure 1). Our ICH and radiology database review was approved by the Ethics Committee of the Second Affiliated Hospital of Bengbu Medical University. The need for consent to participate was waived by the Ethics Committee of the Second Affiliated Hospital of Bengbu Medical University. The data supporting the findings of this study are available from the corresponding author upon reasonable request.

### 2.2. Data Collection and Outcome Measures

**FIGURE 2:**
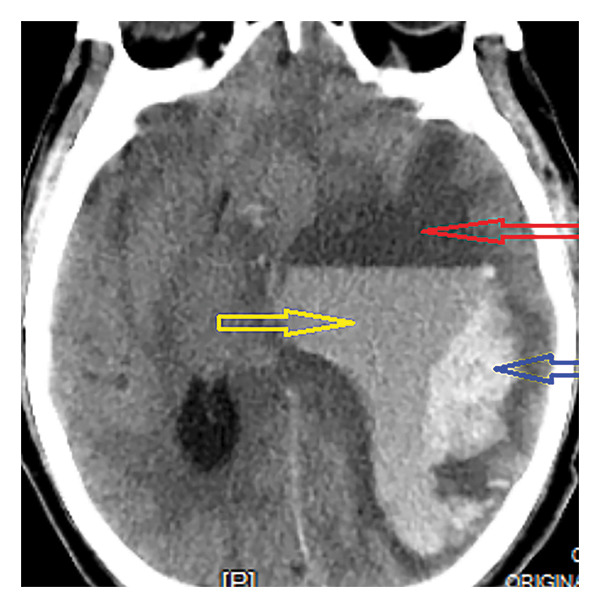
Schematic diagram of the blend and edema signs. The blue arrow locates a dense hematoma. The yellow arrow locates a blend sign with a lower density than the hematoma. The red arrow indicates the edema sign, which is similar to edema brain tissue density but earlier than cerebral edema. It has a large space effect, which influences the prognosis of ICH. Therefore, it is named edema sign.

A retrospective analysis was used in this study, and the enrolled patients were counted and analyzed by the dedicated staff. Medical history data included sex, age, admission systolic and diastolic blood pressure, history of hypertension, coronary heart disease, diabetes mellitus, and history of smoking and alcohol consumption. Disease assessment data included the lowest Glasgow Coma Score (GCS) during treatment, admission venous blood glucose, creatinine, triglyceride, low‐density lipoprotein, the lowest serum albumin concentration during treatment, the lowest total protein during treatment, stress ulceration, cerebral herniation (temporal sulcus herniation), intracranial infection, secondary epilepsy, severe pulmonary infection, the lowest platelets during treatment, the lowest hemoglobin, hematoma volume, hematoma location, and hematoma side, the presence of ventricular casts, hydrocephalus, enlarged hematomas, and the presence of blend sign and edema sign on brain CT (Figure 2). Serum albumin concentration was determined via the bromocresol green (BCG) method. Samples were collected for detection on the 1st, 3rd, 7th, and 14th days postadmission; thereafter (beyond 14 days postadmission), detection was performed based on clinical assessment. The lowest serum albumin level during the entire hospitalization period is selected (excluding abnormal values caused by excessive fluid administration), which serves as the lowest serum albumin level. In addition to the use of intravenous albumin supplementation, the exact amount and cost of its use were also recorded. A prognostic assessment was performed using the mRS. Based on the 90‐day mRS, the patients were divided into the group with a good outcome (mRS ≤ 2, 111 cases) and the group with a poor outcome (mRS > 2, 157 cases).

### 2.3. Statistical Methods

The Kolmogorov–Smirnov or Shapiro–Wilk tests were used to test normality. The homogeneity of variance was tested with Levene’s test. As most outcomes showed a non‐normal distribution and heterogeneity of variance, statistical data are reported as medians with interquartile ranges (IQRs) and compared with the Kruskal–Wallis H test for multiple independent samples and the Mann–Whitney *U* test for two independent samples. Categorical data are shown as frequencies and percentages, and the chi‐squared test was used to compare differences between the two groups. Multifactorial analysis was performed using logistic regression analysis. In addition, the receiver operator characteristic (ROC) curve was used to assess the diagnostic value of prognostic factors influencing the prognosis of patients with ICH. The ROC curve analysis used was the method of DeLong et al.

Given that clinical practice has shown that consciousness level may influence albumin levels, this study employed Spearman correlation analysis to investigate the correlation between human serum albumin and GCS scores and further analyzed albumin differences across different consciousness levels.

All analyses were performed using SPSS Statistics Version 22 (IBM Corp., Armonk, NY, USA) and MedCalc 18.11 (Ostend, Belgium). A *p* value < 0.05 was considered to be statistically significant.

## 3. Results

### 3.1. Factors Influencing the Outcome of Patients With ICH

**FIGURE 3:**
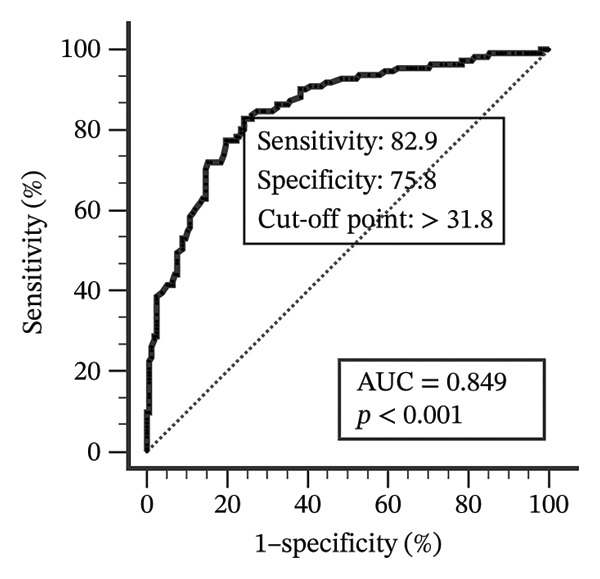
ROC curve of the lowest serum albumin: the lowest serum albumin > 31.8 g/L (AUC = 0.849, *p* < 0.001) predicted a good outcome for patients with ICH.

Univariate analysis identified significant differences between the two groups for age, GCS, hematoma volume, serum albumin concentration, total protein, admission venous glucose, total cholesterol, low‐density lipoprotein, hemoglobin, platelets, cerebral infarction, ventricular casts, hydrocephalus, blend sign, edema sign, severe pulmonary infection, intracranial infection, stress ulcers, and cerebral herniation (*p* < 0.05, Tables 1 and 2). A multivariate analysis then identified a series of independent factors for the prognoses of patients with ICH, including the lowest GCS, hematoma volume, and the lowest serum albumin concentration (*p* < 0.05). According to the results of standardized regression coefficient analysis, the hematoma volume (*B* ^′^ = −0.839) had the most significant effect on the prognosis of cerebral hemorrhage, followed by GCS (*B* ^′^ = 0.690) and serum albumin concentration (*B* ^′^ = 487). In this regard, the hematoma volume was identified as an independent risk factor for the outcome of patients with ICH, while the lowest GCS and the lowest serum albumin concentration were considered protective factors for the prognosis of ICH (Table 3). Further ROC curve analysis showed that the cutoff points for predicting the outcome of patients with ICH were the lowest with serum albumin > 31.8 g/L (AUC = 0.849, 95% CI: 0.801–0.890, *p* < 0.001, Figure 3), GCS > 11 (AUC = 0.878, 95% CI: 0.833–0.915, *p* < 0.001), and hematoma volume ≤ 23.6 mL (AUC = 0.863, 95% CI: 0.815–0.902, *p* < 0.001). The AUC of the ROC curve for each independent influencing factor was greater than 0.8 and close to 0.9, showing a high diagnostic value.

**TABLE 1:** Correlation between characteristics of patients and the outcome of ICH.

	Good outcome group (*n* = 111)	Poor outcome group (*n* = 157)	*Z*	*p*
Age (years)	58 (17)	60 (18.5)	−2.299	0.022^∗^
Admission systolic blood pressure (mmHg)	161 (36)	164 (36.5)	−0.410	0.682
Admission diastolic blood pressure (mmHg)	97 (24.5)	95 (20)	−1.015	0.310
The lowest GCS score (points)	14 (3)	8 (5)	−10.606	< 0.001^∗^
Hematoma volume (mL)	9.7 (13.10)	33.20 (27.95)	−10.097	< 0.001^∗^
Time of onset (hour)	4 (11.50)	4 (6.50)	−0.215	0.830
The lowest serum albumin (g/L)	36.60 (5.95)	29.20 (4.90)	−9.742	< 0.001^∗^
The lowest total protein (g/L)	62.20 (8.00)	53.50 (7.90)	−9.135	< 0.001^∗^
Admission venous blood glucose (mmol/L)	6.86 (2.06)	7.85 (3.94)	−4.366	< 0.001^∗^
Serum creatinine (μmol/L)	54.00 (23.00)	55.00 (25.00)	−0.487	0.626
Triglyceride (mmol/L)	1.41 (0.95)	1.43 (1.19)	−0.174	0.862
Total cholesterol (mmol/L)	4.37 (1.06)	4.13 (1.34)	−2.046	0.041^∗^
Admission LDL (mmol/L)	1.92 (1.00)	1.72 (1.39)	−2.052	0.040^∗^
The lowest hemoglobin (g/L)	124 (20.50)	92 (31.00)	−9.381	< 0.001^∗^
The lowest platelet (10^9^/L)	173 (78.50)	138 (74)	−4.939	< 0.001^∗^

*Note:* The test used was the Mann–Whitney *U* test.

^∗^ *p* < 0.15.

**TABLE 2:** Correlation between characteristics of patients and the outcome of ICH.

	Good outcome group (*n* = 111)	Poor outcome group (*n* = 157)	*χ* ^2^	*p*
Sex, male (%)	71 (63.96)	108 (68.79)	0.683	0.409
Hypertension (%)	80 (72.07)	124 (78.98)	1.707	0.191
Diabetes (%)	9 (8.12)	19 (12.10)	1.109	0.292
Coronary heart disease (%)	3 (2.70)	4 (2.55)	0.006	0.938
Cerebral infarction (%)	7 (6.31)	23 (14.65)	4.553	0.033^∗^
Hematoma site (%)				
Basal ganglia region	65 (58.56)	98 (62.42)	1.516	0.469
Thalamus	21 (18.92)	33 (21.02)		
Lobe	25 (22.52)	26 (16.56)		
Hematoma side, left (%)	59 (53.15)	83 (52.87)	0.002	0.963
Ventricular cast (%)	1 (0.90)	12 (7.64)	6.405	0.011^∗^
Hydrocephalus (%)	2 (1.80)	23 (14.65)	12.690	< 0.001^∗^
Enlarged hematoma (%)	1 (0.90)	8 (5.10)	3.512	0.061^∗^
Blend sign (%)	12 (10.81)	55 (35.03)	20.346	< 0.001^∗^
Edema sign (%)	9 (8.12)	36 (22.93)	10.225	0.001^∗^
History of alcohol consumption (%)	39 (35.14)	53 (33.76)	0.055	0.815
History of smoking (%)	28 (25.23)	50 (31.85)	1.382	0.240
Serious pulmonary infection (%)	11 (9.91)	92 (58.60)	65.147	< 0.001^∗^
Intracranial infection (%)	3 (2.70)	35 (22.29)	20.508	< 0.001^∗^
Stress ulcer (%)	8 (7.21)	42 (26.75)	16.367	< 0.001^∗^
Brain hernia (%)	0 (0.00)	22 (14.01)	16.945	< 0.001^∗^
Secondary epilepsy (%)	0 (0.00)	3 (1.91)	2.137	0.144^∗^

^∗^ *p* < 0.15.

**TABLE 3:** Multivariate analysis of predictors for the prognosis of ICH.

	*B*	*p*	*B* ^′^	Exp (*B*)	95% CI of exp (*B*)	
					Lower limit	Upper limit
The lowest GCS score	0.318	< 0.001	0.690	1.375	1.205	1.569
Hematoma volume (mL)	−0.064	< 0.001	−0.839	0.938	0.911	0.965
The lowest serum albumin (g/L)	0.163	< 0.001	0.487	1.177	1.081	1.281

*Note: B* is the regression coefficient; *B* ^′^ is standardized regression coefficient; Exp (*B*) is the odds ratio (OR).

Abbreviation: CI, confidence interval.

### 3.2. The Relationship Between the Lowest Serum Albumin Concentration and Consciousness in Patients With ICH

**FIGURE 4:**
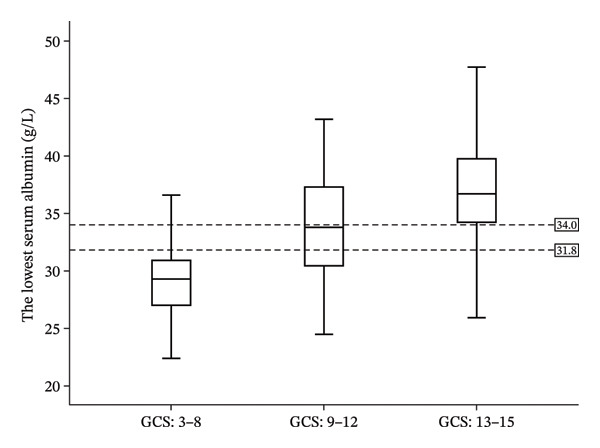
Comparison of the lowest serum albumin between different levels of consciousness: GCS 3–8 subgroup, 9–12 subgroup, and 13–15 subgroup, and the median and IQR of the three groups were 29.30 (3.90), 33.80 (6.85), and 36.70 (5.55), respectively; there was a significant difference between the three groups (*χ* ^2^ = 69.696, *p* < 0.001, Figure 3). Pairwise comparisons also confirmed that there was a significant difference in albumin levels between the groups (*p* < 0.05).

A correlation analysis between the lowest serum albumin concentration and the lowest GCS using Spearman’s rank correlation method showed that the rank correlation coefficient (*r*_*s*_) was 0.569 (*p* < 0.001), indicating that albumin level was positively correlated with the lowest GCS. To further explore this relationship, we divided the patients into three groups according to the lowest GCS: GCS 3–8 subgroup, 9–12 subgroup, and 13–15 subgroup. The median and IQR of the three groups were 29.30 (3.90), 33.80 (6.85), and 36.70 (5.55), respectively. There was a significant difference between the three groups (*χ* ^2^ = 69.696, *p* < 0.001, Figure 4). Pairwise comparisons also confirmed a significant difference in albumin levels between the groups (*p* < 0.05).

## 4. Discussion

As an important component of plasma, albumin has an irreplaceable role in maintaining plasma osmotic pressure [8], microvascular permeability [9], acid–base balance [10], and preventing platelet aggregation [11] and is commonly used clinically to reduce cerebral edema [12]. Hypoalbuminemia (defined as a serum albumin concentration of less than 34 g/L) is commonly seen in critically ill patients and has been shown to be associated with a poorer prognosis, affecting morbidity and mortality in a variety of diseases [13–15]. The Lund concept further demonstrated the important role of serum albumin in neurological disorders [16]. The clinical study of Cao and Yao showed that hypoalbuminemia on admission was a prognostic factor for poor prognosis in patients with ICH [17]. In addition, other studies have demonstrated that age, low GCS on admission, hematoma location, hematoma volume, hematoma enlargement, hydrocephalus, hyperglycemia on admission, renal insufficiency, and a number of serological biomarkers are also independent predictors of poor prognosis in ICH [18–24]. In this study, all these predictors were statistically analyzed, and the results showed that the lowest GCS, the lowest serum albumin concentration, and hematoma volume were independent influences on the prognosis of patients with ICH (*p* < 0.001), with hematoma volume being an independent risk factor for the prognosis of ICH. The lowest GCS and the lowest albumin concentration were protective factors for the prognosis of ICH. Notably, the correlation between the lowest serum albumin concentration and ICH prognosis showed positive results even with albumin supplementation in ICH patients, which further confirmed the important role of serum albumin in the prognosis of ICH.

Previous studies have shown that about 20% of patients with cerebrovascular disease have lower than normal albumin levels in the 4 days after disease onset [25]. Another study by Aptaker et al. also noted that more than half (53%) of patients with cerebrovascular disease treated in rehabilitation wards had individual serum albumin concentrations below the normal range [26]. In this study, 59.33% (159/268) of patients with ICH had serum albumin levels lower than normal (< 34 g/L), 51.49% (138/268) had serum albumin below or equal to 31.8 g/L, and 38.43% (103/268) had serum albumin lower than 30 g/L. From the results of this study, serum albumin > 31.8 g/L (AUC = 0.849, *p* < 0.001) predicted a good outcome for patients with ICH. Therefore, to improve the prognosis of patients with ICH, albumin supplementation would be needed in about 51.49% of patients. In addition, if, according to the Lund concept, serum albumin should be greater than or equal to 40 g/L in order to improve the prognosis of patients with neurocritical illnesses, 89.55% (240/268) of the patients would need to be supplemented with albumin. This would be very difficult to achieve in the clinical setting. Correcting a patient’s serum albumin abnormalities with albumin would be a huge financial burden for the patient’s family, as albumin is expensive and usually not covered by health insurance (unless the condition is critical and the serum albumin level is less than 30 g/L). In this study, a total of 115 (42.91%) patients received albumin supplementation, with a mean of 24.68 supplements per person. The average cost was 11,105.22 yuan. This fully illustrated the heavy economic burden of hypoalbuminemia in the treatment of ICH.

It is often observed in clinical practice that once coma symptoms develop in patients with ICH, serum albumin levels often drop rapidly, often below 30 g/L. In contrast, patients with drowsiness or lethargy, even with a poor diet, were unvulnerable to severe hypoalbuminemia (serum albumin concentration < 30 g/L). In view of this, a thorough analysis of the correlation between serum albumin concentration and GCS was performed in this study. This study’s results indicated a correlation between the degree of impaired consciousness and albumin level. As the degree of impaired consciousness increased, the albumin levels of patients tended to decrease. Specifically, most (more than 75%) albumin levels in patients with mild consciousness disorder or an awake state (GCS 13–15) were in the normal range (≥ 34 g/L). In patients with moderate consciousness disorder (GCS 9–12), although the albumin level was low, most (about 75%) were still higher than 31.8 g/L, which had a lesser impact on the prognosis, and there was no need to additionally supplement the albumin in the clinic. However, in patients with severe consciousness disorder, namely, coma (GCS 3–8), the albumin level was generally (more than 75%) lower than 31.8 g/L. This was a was an independent predictor of poor outcome in ICH, in which case albumin should be actively supplemented to improve the condition according to holistic medicine [27]. Therefore, we know that ICH patients in a coma are the main group requiring clinical albumin supplements.

However, this study is a retrospective study, and our findings represent “correlation” rather than “causation,” especially for the lowest serum albumin and prognosis of patients with ICH. It is unknown whether albumin supplementation affects prognosis. Preclinical studies have shown that albumin‐mediated neuroprotection may stem from its biological functions, including its major antioxidation activity, anti‐inflammatory responses, and antiapoptosis. Human serum albumin treatment can provide neuroprotective and recovery enhancement effects via improving short and long‐term neurologic function, maintaining blood–brain barrier (BBB) integrity and reducing neuronal oxidative stress and apoptosis [17]. Clinical studies have shown albumin evidently improves abnormal EEG in cases with hemorrhage and mass effects in the CT scans, suggesting the action is possibly mediated by lowering intracranial pressure and dehydrating brain edema [28]. Therefore, the supplementation of human albumin may have a positive effect on the prognosis of patients with ICH.

Currently, although there is a study on albumin for cerebral hemorrhage intervention, it was terminated due to poor enrollment. Although surgical intervention is the standard treatment for patients with ICH, its efficacy remains controversial [29–32]. Therefore, future research on albumin therapy for intracerebral ICH is warranted, particularly randomized controlled trials. The results of our study can provide an important reference basis and screening criteria for designing future RCTs to verify albumin efficacy.

Although this study provided valuable insights into the prognostic relevance of serum albumin in ICH, there were some limitations that cannot be ignored. First, this study was limited to a single‐center study with a relatively limited sample size, which may impact the general applicability of the results. Second, this study did not cover all potential confounding variables when considering influencing factors. These variables included the nutritional status of the patients, the status of liver function, and the level of inflammatory markers, which may have an impact on the serum albumin level [33–35]. Finally, this study failed to explore in depth the possible joint effects between albumin levels and other biomarkers, which is a direction that needs to be expanded in subsequent studies.

## 5. Conclusions

According to our findings, this study demonstrates for the first time that the lowest serum albumin level during hospitalization serves as a robust and independent prognostic predictor for ICH. While its causal relationship and clinical value require further validation through prospective studies, this biomarker provides a straightforward and objective tool for risk stratification in ICH patients and future personalized adjuvant therapy research. In addition, the degree of impaired consciousness may have an effect on serum albumin levels, especially in comas. However, the question of why comatose patients develop severe hypoalbuminemia needs to be further explored.

**Nomenclature**
ICH: Intracerebral hemorrhage
GCS: Glasgow Coma Score
mRS: Modified Rankin Scale
IQRs: Interquartile ranges
ROC: Receiver operator characteristic
AOC: Area under the curve

## Author Contributions

Conceptualization: Biao Zhao and Da‐Wei Wang; methodology: Biao Zhao; software: Hua‐Zhen Zhang; validation: Biao Zhao and Da‐Wei Wang; formal analysis: Biao Zhao and Tao Liu; investigation: Hua‐Zhen Zhang and Dan Liu; resources: Da‐Wei Wang; data curation: Hua‐Zhen Zhang; writing–original draft preparation: Hua‐Zhen Zhang and Dan Liu; writing–review and editing: Biao Zhao and Da‐Wei Wang; visualization: Biao Zhao; supervision: Da‐Wei Wang; project administration: Da‐Wei Wang; funding acquisition: Da‐Wei Wang.

## Funding

This research was funded by the University Natural Science Research Project of Anhui Province (Grant no. 2024AH051232).

## Disclosure

Our manuscript is not under consideration in another journal, and we take full responsibility for this statement. The funders had no role in the design of the study; in the collection, analyses, or interpretation of data; in the writing of the manuscript; or in the decision to publish the results. All authors have read and agreed to the published version of the manuscript [36].

## Ethics Statement

The study was conducted in accordance with the Declaration of Helsinki and approved by the Ethics Committee of the Second Affiliated Hospital of Bengbu Medical University (protocol code [2024] KY021 and June 21, 2024).

## Consent

Because this research was a retrospective study, the need for consent to participate was waived by the Ethics Committee of the Second Affiliated Hospital of Bengbu Medical University.

## Conflicts of Interest

The authors declare no conflicts of interest.

## Data Availability

The data supporting the findings of this study are available from the corresponding author upon reasonable request.
